# Orally administered scallop-derived plasmalogens improve cognitive function in healthy middle-aged and older adults: a randomized, double-blind, placebo-controlled trial

**DOI:** 10.3389/fnut.2026.1906996

**Published:** 2026-08-12

**Authors:** Mina Fukuchi, Mizuho Tanaka, Masafumi Nagata, Tsuyoshi Takara, Yasuhiro Sasuga

**Affiliations:** 1 Hachioji Center for Research and Development, B&S Corporation Co. Ltd., Tokyo, Japan; 2 ORTHOMEDICO Inc, Tokyo, Japan; 3 Minami-Machi Clinic, Nerima Medical Association, Tokyo, Japan; 4 Takara Clinic, Medical Corporation Seishinkai, Tokyo, Japan

**Keywords:** cognitive performance, dietary supplementation, plasmalogens, randomized controlled trial, scallop, verbal memory

## Abstract

**Background:**

Plasmalogens (Pls) are ether phospholipids enriched in the brain that help maintain synaptic integrity and support cognitive function. However, evidence of their cognitive benefits in otherwise healthy individuals remains limited. This randomized, double-blind, placebo-controlled trial examined whether supplementation with scallop-derived Pls (sPls) improves cognitive performance in cognitively healthy Japanese adults aged ≥40 years with below-average verbal memory at baseline.

**Methods:**

Of 168 individuals screened, 68 eligible participants were randomly assigned (1:1) to receive either sPls (1 mg/day) or placebo for 12 weeks. Cognitive performance was assessed using the web-based Cognitrax battery (CNS Vital Signs), with the standardized verbal memory score as the primary outcome. Secondary outcomes included other cognitive domains and plasma brain-derived neurotrophic factor (BDNF) levels. Safety was evaluated through adverse event monitoring and clinical assessments.

**Results:**

Sixty-six participants were included in the analysis. After 12 weeks, standardized verbal memory scores were significantly higher in the sPls group than in the placebo group (estimated marginal mean difference, 10.1; 95% confidence interval, 0.4–19.9; *p* = 0.042). The sPls group also showed significantly greater improvements in selected attention-related cognitive domains. Plasma BDNF levels increased in both groups, with no significant between-group differences; a modest negative correlation between BDNF levels and cognitive performance was observed in the sPls group at 12 weeks. No adverse events or clinically meaningful safety concerns were reported.

**Conclusion:**

In cognitively healthy middle-aged and older adults with below-average verbal memory, 12 weeks of sPls supplementation was associated with improved verbal memory compared with placebo. Exploratory analyses also suggested potential improvements in selected attention-related cognitive domains. Dietary sPls may have the potential to support cognitive function in this population; however, further studies are required to confirm these findings and evaluate long-term safety.

**Clinical trial registration:**

https://center6.umin.ac.jp/cgi-open-bin/ctr/ctr_view.cgi?recptno=R000066286, identifier UMIN000057985.

## 1 Introduction

Age-related cognitive decline is an increasing public health concern, affecting not only older adults but also their families and society as a whole. Such cognitive decline can impair everyday abilities—including memory, attention, language, and executive function—and potentially contribute to the development of dementia (1, 2). Among the various cognitive domains, episodic memory tends to show the greatest decline. This has been linked to structural and functional changes in the hippocampus, a brain region essential for encoding and retrieving new information (3, 4). In contrast, semantic memory is relatively well preserved with age and may even improve over time as knowledge and experience accumulate (5, 6).

Cognitive decline arises from a complex interplay of nonmodifiable factors, such as aging, genetic polymorphisms, and family history, as well as modifiable lifestyle factors, including physical inactivity, social isolation, metabolic conditions (e.g., hypertension, hypercholesterolemia, diabetes, and obesity), unhealthy dietary habits, and excessive alcohol use (7, 8). Accordingly, current approaches to maintaining cognitive health extend beyond pharmacological treatments to include lifestyle-based interventions such as regular physical activity and cognitive training. Moreover, there is growing interest in natural products and dietary supplements as accessible options for supporting cognitive function. A range of bioactive compounds, including phosphatidylserine, omega-3 fatty acids, flavonoids, turmeric, *ginkgo biloba* extract, and coffee cherry extract, have been studied for their potential neuroprotective effects and benefits for cognitive performance (9–12).

Plasmalogens (Pls) are dietary ether phospholipids that have recently drawn attention for their potential role in brain health. They belong to a subclass of glycerophospholipids distinguished by a vinyl ether bond at the sn-1 position and are widely distributed in the human brain and other tissues (13). Maintaining adequate levels of Pls appears to be important for normal central nervous system and vascular function, and growing evidence suggests that reduced levels may contribute to the development of neurodegenerative diseases (14, 15). In animal studies, Pls administration has been shown to suppress inflammation-related microglial activation, reduce amyloid deposition and neuronal loss, and improve cognitive performance (16–18).

Clinical studies have also pointed to possible cognitive benefits of Pls supplementation in disease settings. In particular, purified scallop-derived Pls (sPls) have been reported to improve cognitive function in patients with mild Alzheimer’s disease (AD) or mild cognitive impairment (MCI) (19, 20). Benefits have also been observed in individuals with more advanced AD and in patients with Parkinson’s disease (21, 22). However, in studies involving MCI populations, cognitive outcomes were largely assessed using subscales of the Mini-Mental State Examination-Japanese (MMSE-J), a screening tool intended to detect dementia rather than capture subtle cognitive changes (20). As a result, the effects of sPls on cognitive function in cognitively healthy individuals remain unclear.

More broadly, recent studies have continued to emphasize the potential importance of nutritional factors in cognitive function, further supporting investigation of dietary approaches to cognitive health (23). From a preventive perspective, it is important to understand whether sPls supplementation can support cognitive function in healthy adults before any clear clinical impairment develops. To address this, we conducted a randomized, placebo-controlled study to evaluate the effects of sPls on cognitive function in healthy Japanese adults, using a comprehensive computerized assessment battery (the Japanese version of CNS Vital Signs, Cognitrax) (24, 25). Plasma brain-derived neurotrophic factor (BDNF), a neurotrophin involved in learning, memory, and neurogenesis, and potentially linked to Pls-related mechanisms of cognitive function, was also measured as an exploratory biomarker (18).

## 2 Materials and methods

### 2.1 Study design and ethical approval

This study was a randomized, double-blind, placebo-controlled trial with a parallel-group design and a 1:1 allocation ratio. Efficacy and safety were assessed at baseline and after 12 weeks of intervention (Table 1). It was conducted in accordance with the Declaration of Helsinki and the Ethical Guidelines for Medical and Biological Research Involving Human Subjects in Japan. The study protocol was approved by the Ethics Committee of Takara Clinic, Medical Corporation Seishinkai, on May 13, 2025 (Approval ID: 2505–01248-0029-10-TC). The trial was registered with the University Hospital Medical Information Network Clinical Trials Registry on May 27, 2025 (UMIN000057985, https://center6.umin.ac.jp/cgi-open-bin/ctr/ctr_view.cgi?recptno=R000066286) under the title “Effects of consumption of the test food on cognitive function: a randomized, placebo-controlled, double-blind, parallel-group comparison study.” No major changes to the protocol were made after the study began.

**Table 1:** Schedule of the study.

Study phase	Item	Study period				
		Enrollment	Screening (Baseline)	Allocation	Post-allocation	
					Start intake	12 weeks
Registration	Eligibility screening	●				
	Informed consent	●				
	Allocation			●		
Interventions	sPls group				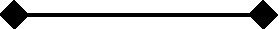	
	Placebo group				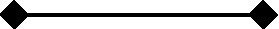	
Assessment	Cognitrax test		●			●
	Dietary survey		●			●
	Physical examination		●			●
	Urinalysis		●			●
	Blood test		●			●
	MMSE-J		●			●
	Daily record		●		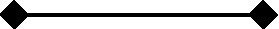	
	Medical questionnaire		●			●

“●” indicates assessment or procedure performed at the specified time point. “◆” indicates daily intervention or activity throughout the study period. sPls, scallop-derived plasmalogens; MMSE-J, Mini-Mental State Examination-Japanese.

### 2.2 Participants

Inclusion criteria were healthy Japanese adults, both sexes, aged ≥40 years with verbal memory performance below the age-matched normative mean, absence of clinically diagnosed disease, a MMSE-J score of at least 24 at screening and a validity index of “yes” on the Cognitrax verbal memory test with a standardized verbal memory score <100 at screening. All participants provided written informed consent.

Exclusion criteria included serious medical conditions; implanted cardiac devices; chronic disorders that could affect cognition; diagnosed dementia; irregular sleep patterns; frequent use of foods or supplements known to influence cognitive function; shellfish allergy; pregnancy or lactation; participation in another clinical trial within the past 28 days, or any other reason deemed appropriate by the investigator.

Participants were recruited through an online registry managed by ORTHOMEDICO Inc. (Tokyo, Japan). All study procedures and data collection were conducted at Takara Clinic.

### 2.3 Sample size

Because no previous studies had evaluated changes in verbal memory using Cognitrax after 12 weeks of sPls supplementation, we assumed a large between-group effect size of Cohen’s *d* = 0.80 (26). With a two-sided significance level of 0.05 and 80% power, a minimum of 52 participants (26 per group) was required. To increase statistical power within budget constraints, we set the target sample size at 60 participants (30 per group). Allowing for an anticipated dropout rate of about 10%, the final planned sample size was increased to 68 participants (34 per group).

### 2.4 Selection, randomization, and blinding

Of the 168 individuals who provided informed consent, 68 met the eligibility criteria and were randomly assigned to the sPls or placebo group. Randomization was performed using stratified block method based on age, sex, and baseline standardized verbal memory score. An independent administrator generated the allocation sequence using a computer-generated randomization program (R software version 4.5.1, blockrand package version 1.5). Participants, investigators, and study staff remained blinded to group assignments until the database was locked. To minimize potential bias, participant recruitment, clinical assessments, and laboratory analyses were conducted by organizations independent of the study sponsor.

### 2.5 Intervention

Participants were instructed to take two capsules (sPls or placebo) once daily with water before breakfast for 12 weeks. Each sPls capsule contained 0.5 mg of purified sPls extracted from *Mizuhopecten yessoensis*, consistent with previous studies (19–22, 27), for a total daily dose of 1.0 mg. Placebo capsules were identical in appearance and composition but did not contain sPls. Adherence was assessed by counting returned capsules.

The dosage was determined based on previous studies reporting cognitive benefits with sPls supplementation (19–21) as well as studies using Pls derived from non-scallop sources (28, 29).

### 2.6 Primary outcomes

The primary outcome was the standardized verbal memory score obtained from the web-based Cognitrax battery (Health Solution, Inc., Tokyo, Japan), based on CNS Vital Signs, measured at 12 weeks (24, 25). Assessments were conducted at baseline and at the end of the 12-week intervention period.

The Cognitrax battery includes 10 tests (verbal memory, visual memory, finger tapping, symbol digit coding, stroop test, shifting attention, continuous performance, perception of emotions, non-verbal reasoning, and the four-part continuous performance test). These tests generate scores across 16 cognitive domains: neurocognitive index, composite memory, verbal memory, visual memory, psychomotor speed, reaction time, complex attention, cognitive flexibility, processing speed, executive function, social acuity, reasoning, working memory, sustained attention, simple attention, and motor speed. Raw scores were normalized against an age-matched reference population to produce standardized scores, with higher scores indicating better performance.

### 2.7 Secondary outcomes

Cognitive domains other than verbal memory were analyzed using both standardized and raw Cognitrax scores.

### 2.8 Exploratory outcomes

Exploratory outcomes included plasma BDNF levels and subgroup analyses of the standardized verbal memory score stratified by educational attainment. Plasma BDNF levels were measured at baseline and at 12 weeks to explore potential associations with cognitive performance. Blood samples were collected at baseline and after 12 weeks to measure plasma BDNF levels. Analyses were conducted at LSI Medience Corporation (Tokyo, Japan), along with additional laboratory tests for safety monitoring.

An exploratory subgroup analysis of standardized verbal memory scores was performed based on educational attainment.

In addition, the association between plasma BDNF levels and standardized verbal memory scores was evaluated using correlation analyses.

These analyses were not powered *a priori* and were performed for hypothesis generation.

### 2.9 Safety evaluation

Safety was assessed by monitoring adverse events and conducting physical examinations, urinalysis, and blood tests, including liver and renal function, blood glucose, and lipid profiles, at each study visit. Adverse event reporting, physical measurements, and sample collection were performed at Takara Clinic. Urine and blood analyses were performed by LSI Medience Corporation using standard laboratory methods.

### 2.10 Statistical analysis

The full analysis set (FAS) comprised all randomized participants who received at least one dose of the study intervention and had evaluable data at both baseline and 12 weeks. All primary, secondary, and safety analyses were based on the FAS. An exploratory subgroup analysis of standardized verbal memory scores was performed based on educational attainment, with participants categorized according to whether they had attained a bachelor’s degree.

Categorical variables are presented as counts and percentages, and continuous variables are presented as mean ± standard deviation (SD). Between-group comparisons at baseline were performed using the chi-square test for categorical variables and Welch’s t-test for continuous variables. Within-group changes from baseline to 12 weeks were assessed using paired t-tests with false discovery rate correction (30).

Between-group differences at 12 weeks were evaluated using analysis of covariance (ANCOVA), with baseline values included as a covariate and treatment group included as a fixed factor. Estimated marginal means (EMMs) with standard errors (SEs), between-group differences, and corresponding 95% confidence intervals (CIs) were calculated. Effect sizes (Cohen’s *d*) were calculated using the difference in EMMs divided by the pooled standard deviation at baseline. Associations between plasma BDNF levels and standardized verbal memory scores were examined using Pearson’s correlation coefficient, supported by graphical inspection.

All statistical analyses were two-tailed, with a significance level set at 5%. Analyses were conducted using IBM SPSS Statistics for Windows (version 23.0; IBM Japan Ltd., Tokyo, Japan) and BellCurve for Excel (version 4.10; Addinsoft, Social Survey Research Information Co., Ltd., Tokyo, Japan). Because the primary outcome was prespecified, no adjustments were made for multiple comparisons of secondary or exploratory outcomes. Accordingly, analyses of secondary and exploratory outcomes were considered hypothesis-generating and interpreted cautiously.

## 3 Results

### 3.1 Participant disposition and baseline

**Figure 1:**
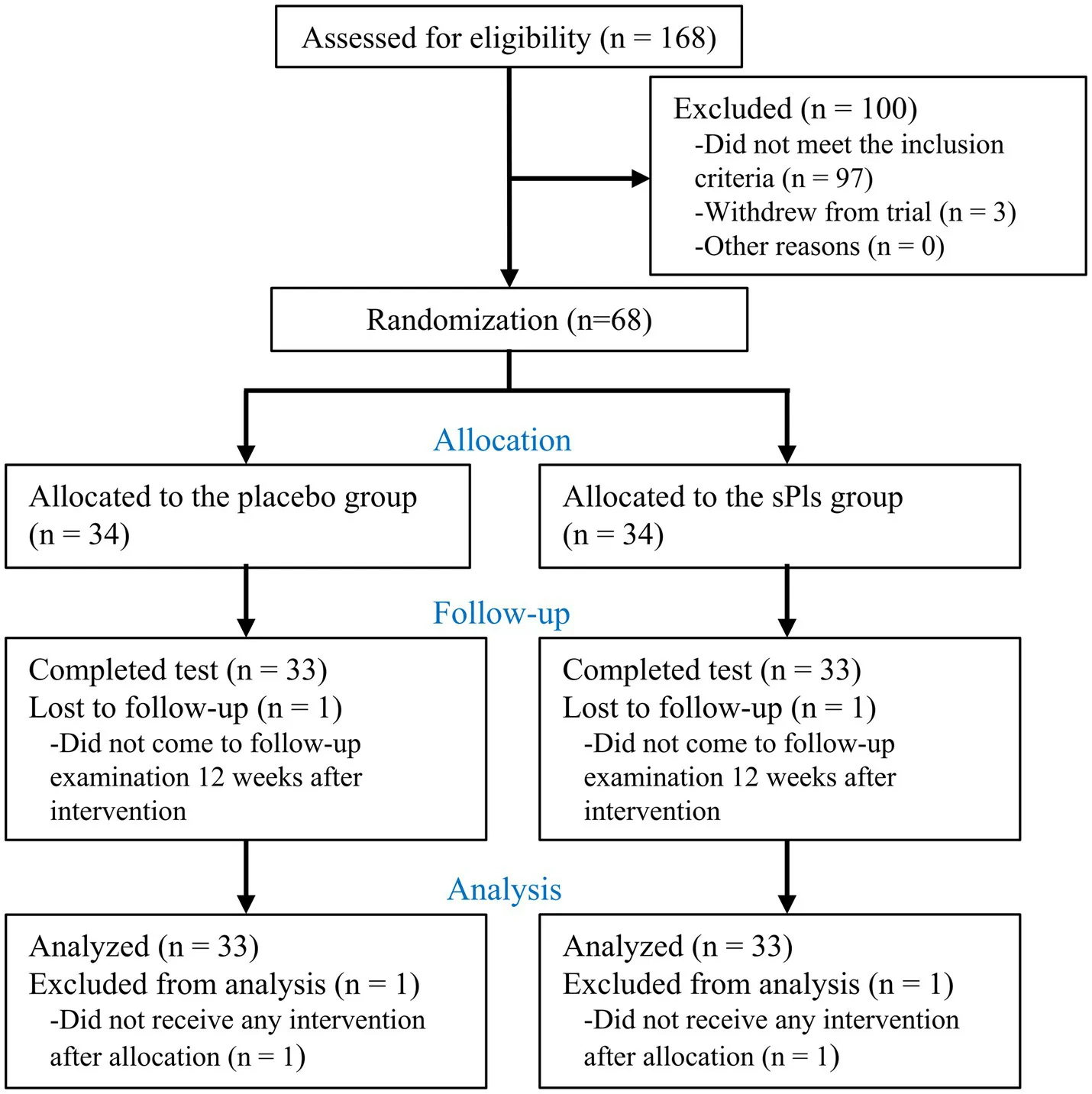
Flow diagram of participant recruitment, allocation, follow-up, and analysis.

Figure 1 presents the flow of participants through the study. Recruitment took place from May 21 to July 11, 2025, and the study ran from June 26 to December 10, 2025. Of the 168 individuals screened, 100 were excluded because they did not meet the eligibility criteria. The remaining 68 participants were randomly assigned to the sPls group (*n* = 34) or the placebo group (*n* = 34). Two participants—one in each group—did not receive the intervention after allocation and were therefore excluded from the analysis. As a result, 66 participants (33 per group) were included in both efficacy and safety analyses based on FAS.

The two groups were generally comparable at baseline (Table 2). Adherence to the intervention was high in both groups, with a mean intake rate exceeding 99.7% in each group. All participants had MMSE-J scores of ≥24, with mean scores of 28.5 ± 1.1 in the placebo group and 28.6 ± 1.3 in the sPls group. Standardized verbal memory scores at baseline were below the age-adjusted average in both groups.

**Table 2:** Baseline characteristics of study participants.

Variables	Placebo (*n* = 33)	sPls (*n* = 33)	*p*-value
Sex, Male *n* (%)/Female *n* (%)	8 (24.2)/25 (75.8)	13 (39.4)/20 (60.6)	0.186
Age, years	53.0 ± 8.7	52.8 ± 8.3	0.945
Height, cm	162.7 ± 8.1	164.9 ± 9.7	0.335
Body weight, kg	55.1 ± 10.1	56.1 ± 8.1	0.650
Body mass index, kg/m^2^	20.7 ± 2.9	20.5 ± 1.5	0.800
Systolic blood pressure, mmHg	113.2 ± 15.9	111.0 ± 12.7	0.528
Diastolic blood pressure, mmHg	72.5 ± 11.5	71.9 ± 8.5	0.808
MMSE-J	28.5 ± 1.1	28.6 ± 1.3	0.610
Verbal memory score (standardized)	78.6 ± 17.0	79.9 ± 14.9	0.742
Educational attainment			
Middle school, *n* (%)	0 (0.0)	0 (0.0)	0.522
High school, *n* (%)	9 (27.3)	4 (12.1)	
Vocational/technical school, *n* (%)	3 (9.1)	4 (12.1)	
Associate degree, *n* (%)	8 (24.2)	8 (24.2)	
Bachelor’s degree, *n* (%)	12 (36.4)	14 (42.4)	
Graduate degree (Master’s/Doctoral), *n* (%)	1 (3.0)	3 (9.1)	

Categorical variables (sex and educational status) are presented as number (percentage), and continuous variables as mean ± SD. Baseline group comparisons were performed using the chi-square test for categorical variables and Welch’s *t*-test for continuous variables. Educational attainment was self-reported. SD, standard deviation; sPls, scallop-derived plasmalogen; MMSE-J, Mini-Mental State Examination-Japanese.

### 3.2 Primary outcome

Standardized Cognitrax scores are presented in Table 3. The primary outcome, standardized verbal memory score, was significantly higher in the sPls group than in the placebo group at 12 weeks. The EMMs were 89.4 ± 3.5 (SE) in the placebo group and 99.5 ± 3.5 in the sPls group, corresponding to a between-group difference of 10.1 (95% CI, 0.4–19.9; *p* = 0.042). The adjusted between-group effect size (Cohen’s *d*) was approximately 0.63, suggesting a moderate effect. Similar results were observed for raw verbal memory scores (Supplementary Table S1).

**Table 3:** Standardized Cognitrax scores at baseline and 12 weeks.

Outcome	Time point	Placebo (*n* = 33)	sPls (*n* = 33)	Difference (SE)	95% CI	*p*-value	Cohen’s *d*
Neurocognitive index	Baseline	95.9 ± 9.9	98.1 ± 10.6	—	—	0.385	—
	12 weeks	99.8 ± 9.0	103.1 ± 7.9**	1.9 (1.5)	[−1.1, 4.9]	0.202	0.19
Composite memory	Baseline	82.0 ± 16.0	83.7 ± 13.4	—	—	0.636	—
	12 weeks	90.1 ± 19.8	98.1 ± 17.0**	7.3 (4.3)	[−1.4, 15.9]	0.099	0.49
Verbal memory (Primary outcome)	Baseline	78.6 ± 17.0	79.9 ± 14.9	—	—	0.742	—
	12 weeks	89.2 ± 21.6	99.7 ± 18.9***	10.1 (4.9)	[0.4, 19.9]	0.042*	0.63
Visual memory	Baseline	92.2 ± 13.1	93.7 ± 13.1	—	—	0.647	—
	12 weeks	95.1 ± 17.0	97.1 ± 14.7	1.5 (3.8)	[−6.0, 9.1]	0.688	0.11
Psychomotor speed	Baseline	98.8 ± 16.5	104.6 ± 10.9	—	—	0.095	—
	12 weeks	103.0 ± 15.2	106.3 ± 11.8	−1.4 (2.0)	[−5.3, 2.6]	0.492	−0.10
Reaction time	Baseline	100.7 ± 13.3	99.4 ± 22.3	—	—	0.769	—
	12 weeks	103.8 ± 11.4	98.9 ± 11.1	−4.4 (2.3)	[−9.1, 0.2]	0.063	−0.24
Complex attention	Baseline	99.2 ± 17.1	101.8 ± 18.2	—	—	0.551	—
	12 weeks	100.5 ± 10.3	107.2 ± 14.9	5.4 (2.4)	[0.6, 10.3]	0.028*	0.31
Cognitive flexibility	Baseline	98.7 ± 12.3	100.8 ± 14.8	—	—	0.535	—
	12 weeks	102.0 ± 12.0	104.9 ± 15.6	1.2 (2.2)	[−3.1, 5.5]	0.579	0.09
Processing speed	Baseline	110.0 ± 14.1	108.5 ± 10.1	—	—	0.625	—
	12 weeks	112.8 ± 16.1	110.1 ± 9.7	−1.6 (2.4)	[−6.4, 3.1]	0.494	−0.13
Executive function	Baseline	99.0 ± 12.1	100.4 ± 14.9	—	—	0.665	—
	12 weeks	102.8 ± 12.3	104.6 ± 15.5	0.6 (2.2)	[−3.7, 5.0]	0.778	0.04
Social acuity	Baseline	89.5 ± 19.0	83.7 ± 19.7	—	—	0.226	—
	12 weeks	89.5 ± 18.0	86.3 ± 18.7	−1.9 (4.4)	[−10.7, 6.9]	0.665	−0.10
Reasoning	Baseline	101.8 ± 13.4	95.8 ± 15.2	—	—	0.097	—
	12 weeks	98.0 ± 15.7	99.4 ± 16.9	5.4 (3.3)	[−1.3, 12.0]	0.110	0.38
Working memory	Baseline	102.9 ± 11.1	104.1 ± 12.8	—	—	0.698	—
	12 weeks	105.6 ± 8.7	105.7 ± 12.0	−0.3 (2.3)	[−5.0, 4.4]	0.894	−0.03
Sustained attention	Baseline	104.1 ± 11.3	102.9 ± 13.5	—	—	0.709	—
	12 weeks	108.3 ± 5.4	105.0 ± 15.9	−2.8 (2.7)	[−8.1, 2.5]	0.292	−0.22
Simple attention	Baseline	84.5 ± 49.2	92.2 ± 32.0	—	—	0.473	—
	12 weeks	94.1 ± 13.9	100.5 ± 11.1	6.4 (3.1)	[0.1, 12.6]	0.046*	0.15
Motor speed	Baseline	91.5 ± 17.4	100.3 ± 13.0	—	—	0.024*	—
	12 weeks	95.1 ± 13.2	101.5 ± 13.0	0.4 (2.0)	[−3.6, 4.5]	0.828	0.03

Values are mean ± SD. Differences are expressed as sPls minus placebo. Between-group differences were estimated using analysis of covariance (ANCOVA) with baseline values as covariates. Baseline *p*-values were calculated using Welch’s *t*-test. Within-group comparisons were performed using paired *t*-tests. Effect sizes (Cohen’s *d*) were calculated based on adjusted between-group differences.” SD, standard deviation; SE, standard error; CI, confidence interval; sPls, scallop-derived plasmalogens. *p < 0.05, **p < 0.01, ***p < 0.001.

### 3.3 Secondary outcomes

At 12 weeks, the sPls group showed significantly higher standardized scores than the placebo group in complex attention and simple attention (Table 3). The EMM for complex attention was 101.1 ± 1.7 in the placebo group and 106.5 ± 1.7 in the sPls group (difference, 5.4; 95% CI, 0.6–10.3; *p* = 0.028). For simple attention, the corresponding values were 94.1 ± 2.2 and 100.5 ± 2.2 (difference, 6.4; 95% CI, 0.1–12.6; *p* = 0.024). Raw simple attention scores also increased significantly more in the sPls group (Supplementary Table S1).

The composite memory score (the sum of verbal and visual memory scores) was higher in the sPls group, although the difference did not reach statistical significance (*p* = 0.099).

### 3.4 Subgroup analysis by educational attainment

Given the known association between educational attainment and cognitive performance (31), we conducted an exploratory subgroup analysis stratified by educational attainment (Table 4). Among participants with less than a bachelor’s degree, the standardized verbal memory score at 12 weeks was significantly higher in the sPls group than in the placebo group (difference, 19.2; 95% CI, 6.6–31.8; *p* = 0.004). No significant between-group difference was observed among participants with at least a four-year university degree.

**Table 4:** Standardized verbal memory scores stratified by educational attainment.

Subgroup	Time point	Placebo	sPls	Difference (SE)	95% CI	*p*-value
≥ Bachelor’s degree	Baseline	82.0 ± 15.0 (*n* = 13)	81.1 ± 14.7 (*n* = 17)	—	—	0.873
	12 weeks	93.5 ± 22.4 (*n* = 13)	93.2 ± 17.8 (*n* = 17)*	−0.1 (7.5)	[−15.4, 15.2]	0.984
< Bachelor’s degree	Baseline	76.4 ± 18.2 (*n* = 20)	78.6 ± 15.5 (*n* = 16)	—	—	0.695
	12 weeks	86.4 ± 21.2 (*n* = 20)*	106.6 ± 18.2 (*n* = 16)***	19.2 (6.2)	[6.6, 31.8]	0.004**

Values are mean ± SD. Differences are expressed as sPls minus placebo. Between-group differences were estimated using analysis of covariance (ANCOVA) with baseline values as covariates. Baseline *p*-values were calculated using Welch’s *t*-test. Within-group comparisons were performed using paired *t*-tests. Educational attainment was categorized using completion of bachelor’s degree as the cutoff. SD, standard deviation; SE, standard error; CI, confidence interval; sPls, scallop-derived plasmalogens. *p < 0.05, **p < 0.01, ***p < 0.001.

### 3.5 Plasma BDNF

No significant between-group difference in plasma BDNF levels was observed at 12 weeks (Table 5). Plasma BDNF levels increased significantly from baseline in both the placebo group (*p* = 0.005) and the sPls group (*p* = 0.002).

**Table 5:** Plasma BDNF levels at baseline and 12 weeks.

Outcome	Time point	Placebo (*n* = 33)	sPls (*n* = 33)	Difference (SE)	95% CI	*p*-value
Plasma BDNF (pg/mL)	Baseline	1390.3 ± 561.6	1222.9 ± 603.2	—	[−454.0, 119.3]	0.248
	12 weeks	1740.8 ± 646.5**	1528.7 ± 588.0**	−108.3 (125.7)	[−359.5, 142.9]	0.392

Values are mean ± standard deviation (SD). Differences are expressed as sPls minus placebo. Between-group differences were estimated using analysis of covariance (ANCOVA) with baseline values as covariates. Baseline *p*-values were calculated using Welch’s *t*-test. Within-group comparisons were performed using paired *t*-tests. BDNF, brain-derived neurotrophic factor; SD, standard deviation; SE, standard error; CI, confidence interval; sPls, scallop-derived plasmalogens. **p < 0.01.

### 3.6 Correlation between plasma BDNF and verbal memory score

**Figure 2:**
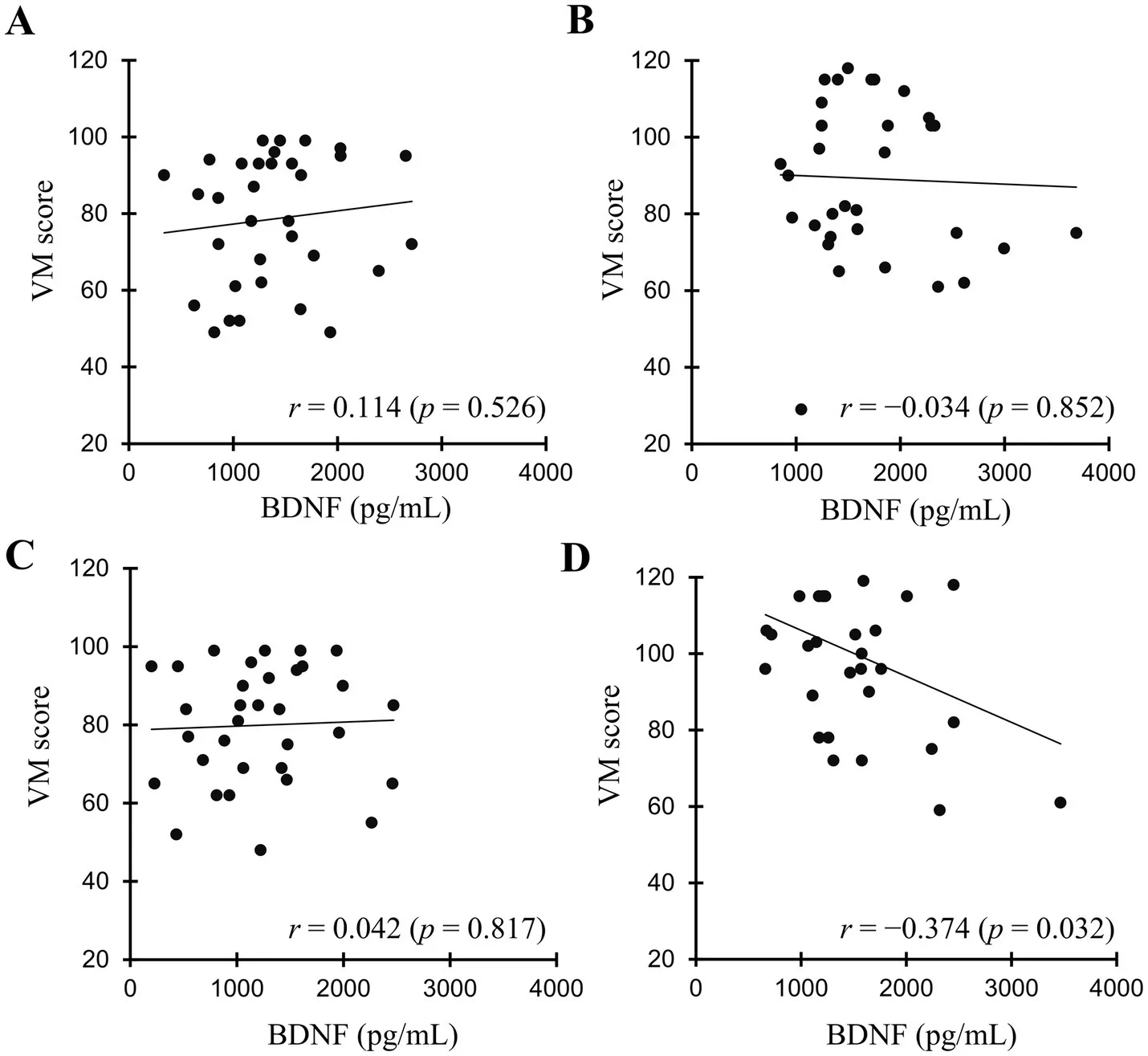
Scatter plots showing correlations between plasma BDNF levels and standardized verbal memory scores assessed by Cognitrax: **(A)** Placebo group at baseline; **(B)** Placebo group at 12 weeks; **(C)** sPls group at baseline; and **(D)** sPls group at 12 weeks.

In the placebo group, plasma BDNF levels were not significantly correlated with standardized verbal memory scores at either baseline or 12 weeks (Figures 2A,B). Similarly, no significant correlation was observed in the sPls group at baseline (Figure 2C). A modest negative correlation was observed in the sPls group at 12 weeks (*r* = −0.374, *p* = 0.032; Figure 2D). Negative correlations were also observed between plasma BDNF levels and working memory (*r* = −0.440, *p* = 0.010) and sustained attention scores (*r* = −0.478, *p* = 0.005) in the sPls group at 12 weeks (Supplementary Table S2).

### 3.7 Safety evaluation

No adverse events were reported in either group. No clinically significant differences in laboratory or urinalysis findings were observed between groups (Supplementary Table S3). Although only total cholesterol (included within lipid metabolism markers) exceeded the reference range more frequently in the sPls group than in the placebo group, this difference was not clinically meaningful. Overall, no medically relevant changes attributable to the intervention were identified based on individual- and group-level safety assessments.

## 4 Discussion

In this randomized, double-blind, placebo-controlled trial, participants receiving sPls showed higher standardized verbal memory scores after 12 weeks compared with those receiving placebo. The estimated between-group difference was approximately 10 points, corresponding to a moderate effect size. However, the primary outcome was associated with a relatively wide confidence interval and a *p*-value close to the conventional threshold for statistical significance. Therefore, although the findings provide evidence supporting a potential benefit of sPls supplementation on verbal memory, the results should be interpreted with appropriate caution and confirmed in larger studies.

Nevertheless, the observed effect may be clinically relevant. Although participants had normal MMSE-J scores, verbal memory performance was below the age-adjusted average at baseline. The observed between-group difference of approximately 10 points shifted the mean verbal memory score in the sPls group from below-average performance toward the normative average range of the Cognitrax classification (25), whereas the placebo group remained below this threshold. Given that verbal memory is a fundamental cognitive domain supporting everyday communication and learning and is known to decline with age (32, 33), these findings may represent a meaningful benefit for individuals with relatively low baseline verbal memory. However, the extent to which this change translates into improved daily functioning remains uncertain.

In addition, repeated administration of the Cognitrax battery may have introduced practice effects. Signals of improvement were observed across multiple cognitive domains in both groups, suggesting that familiarity with the testing procedures contributed to performance gains. Nevertheless, the significant between-group difference in the primary outcome suggests that practice effects alone are unlikely to fully explain the effect of sPls supplementation.

Exploratory analyses also suggested possible improvements in selected attention-related cognitive domains, including complex attention and simple attention. Because multiple secondary outcomes were evaluated without formal adjustment for multiplicity, these findings should be interpreted as hypothesis-generating rather than confirmatory. Attention and verbal memory are closely linked, as efficient encoding and retrieval of verbal information depend on adequate attentional resources (32). Therefore, improvements in attention may have contributed, at least in part, to the observed gains in verbal memory, although the present study was not designed to establish this relationship.

Pls have been reported to support neuronal survival and synaptic function, partly through mechanisms involving BDNF signaling (18). Therefore, plasma BDNF was included as an exploratory biomarker in this study. Although BDNF levels increased significantly from baseline in both groups, no significant difference was observed between the sPls and placebo groups after 12 weeks.

Additionally, correlation analyses revealed a modest negative association between plasma BDNF levels and cognitive performance in the sPls group at 12 weeks, whereas no such relationship was observed in the placebo group. This unexpected finding suggests that the relationship between circulating BDNF and cognitive performance may be more complex than a simple positive association. Therefore, at least based on the findings of the present study, circulating BDNF levels should not be regarded as a direct indicator of cognitive improvement or interpreted as a simple surrogate marker of cognitive enhancement.

Notably, peripheral BDNF concentrations may not accurately reflect central BDNF activity or TrkB signaling in the brain. This limitation may partly explain the absence of a clear relationship between circulating BDNF levels and cognitive outcomes.

In addition, mechanisms other than BDNF signaling, including effects on neuronal membrane integrity, synaptic function, and neuroinflammatory pathways, may contribute to the cognitive effects of sPls. Accordingly, the relationship between sPls supplementation, BDNF dynamics, and cognitive function remains to be clarified using more direct mechanistic biomarkers.

Educational attainment is widely recognized as an important determinant of cognitive reserve, potentially influencing the sensitivity with which intervention-related cognitive changes can be detected (31). In the exploratory subgroup analysis stratified by educational attainment, a significant between-group difference in standardized verbal memory scores at 12 weeks was observed among participants with lower educational attainment, whereas no such difference was observed among those with higher educational attainment. However, this exploratory analysis was not included in the sample size calculation, and the study was not powered to evaluate treatment-by-education interactions. Therefore, no firm conclusions can be drawn regarding differential treatment effects according to educational attainment. These findings should be regarded as hypothesis-generating and should be interpreted cautiously pending replication.

Although Pls derived from different sources may vary in molecular species and fatty acid composition, previous studies using non-scallop-derived Pls have reported improvements in memory function in older adults with MCI, as well as enhancements in subjective cognitive domains such as language, spatial ability, and orientation, along with improvements in overall verbal and visual memory in healthy middle-aged and older adults (28, 29). Accordingly, the present findings should be interpreted in the context of the specific sPls used in this study, while remaining broadly consistent with prior evidence supporting a role of Pls in memory-related cognitive function. Further studies are warranted to clarify both shared and source-specific effects of different Pls preparations on verbal memory. Overall, the present findings add to the growing literature on nutritional approaches to support cognitive health.

Several limitations of this study should also be acknowledged. Although the randomized, double-blind, placebo-controlled design strengthens the internal validity of the study, the sample size was primarily calculated based on the primary outcome. In addition, the sample size calculation was based on an assumed large treatment effect because no prior Cognitrax-based intervention study of sPls was available at the time of study design. If the true treatment effect is smaller than anticipated, the study may have been underpowered to detect modest effects, particularly for secondary cognitive outcomes, educational subgroup analyses, and exploratory biomarker assessments. Consequently, findings beyond the prespecified primary endpoint should be regarded as exploratory and interpreted with appropriate caution.

Another limitation is that repeated administration of the Cognitrax battery may have introduced practice effects. The study was also not powered to evaluate potential treatment differences according to sex or age. Finally, the intervention period was limited to 12 weeks, and the study population consisted of cognitively healthy Japanese adults aged 40 years or older with relatively low baseline verbal memory recruited through an online registry. These characteristics may limit the generalizability of the findings to other ethnic or cultural populations, individuals with average cognitive performance, or patients with cognitive impairment. Additionally, the exceptionally high adherence observed in this trial may not fully reflect real-world settings. Therefore, further studies with longer follow-up periods, larger sample sizes, and more diverse populations are warranted.

## 5 Conclusion

In cognitively healthy middle-aged and older adults with below-average baseline verbal memory, 12 weeks of supplementation with sPls was associated with improved verbal memory compared with placebo. Exploratory analyses also suggested potential improvements in selected attention-related cognitive domains; however, these findings require confirmation in larger studies. Overall, dietary sPls may have the potential to support cognitive function in this population, although further studies are needed to confirm these findings, establish their generalizability, and evaluate long-term safety.

## Data Availability

The raw data supporting the conclusions of this article will be made available by the authors, without undue reservation.
