# Supplementary material for: Orally administered scallop-derived plasmalogens improve cognitive function in healthy middle-aged and older adults: a randomized, double-blind, placebo-controlled trial
Source: Front Nutr. 2026 Aug 12;13:1906996. doi: 10.3389/fnut.2026.1906996 (PMC13506472; doi:10.3389/fnut.2026.1906996)
Supplement: Supplementary file 1 [file Table_1.docx]

**Supplementary Table S1** Raw Cognitrax scores at baseline and 12 weeks

| **Outcome** | **Time point** | **Placebo (n = 33)** | **sPls (n = 33)** | **Difference (SE)** | **95% CI** | ***p*-value** |
| --- | --- | --- | --- | --- | --- | --- |
| Neurocognitive index | Baseline | NA | NA | — | — | NA |
|  | 12 weeks | NA | NA | NA | NA | NA |
| Composite memory | Baseline | 87.5 ± 8.0 | 88.3 ± 7.3 | — | — | 0.678 |
|  | 12 weeks | 91.6 ± 10.1 | 95.7 ± 9.6** | 3.7 (2.3) | [−0.9, 8.2] | 0.110 |
| Verbal memory | Baseline | 45.1 ± 5.1 | 45.4 ± 4.9 | — | — | 0.806 |
|  | 12 weeks | 48.3 ± 6.7 | 51.6 ± 6.4*** | 3.2 (1.6) | [0.1, 6.3] | 0.044* |
| Visual memory | Baseline | 42.5 ± 4.4 | 43.0 ± 4.4 | — | — | 0.658 |
|  | 12 weeks | 43.4 ± 5.7 | 44.1 ± 5.2 | 0.6 (1.3) | [−2.0, 3.1] | 0.666 |
| Psychomotor speed | Baseline | 160.7 ± 24.3 | 169.1 ± 18.3 | — | — | 0.117 |
|  | 12 weeks | 166.5 ± 20.2 | 171.5 ± 17.0 | −1.4 (2.4) | [−6.1, 3.4] | 0.567 |
| Reaction time | Baseline | 669.7 ± 101.9 | 677.5 ± 154.3 | — | — | 0.810 |
|  | 12 weeks | 651.1 ± 91.3 | 675.8 ± 79.5 | 21.4 (16.3) | [−11.1, 54.0] | 0.193 |
| Complex attention | Baseline | 8.3 ± 7.9 | 6.8 ± 6.0 | — | — | 0.363 |
|  | 12 weeks | 7.3 ± 3.8 | 5.0 ± 5.2 | −1.7 (0.9) | [−3.5, 0.1] | 0.064 |
| Cognitive flexibility | Baseline | 40.8 ± 12.8 | 43.0 ± 12.5 | — | — | 0.486 |
|  | 12 weeks | 43.7 ± 11.0 | 46.2 ± 13.3 | 0.9 (1.9) | [−3.0, 4.7] | 0.659 |
| Processing speed | Baseline | 55.9 ± 10.7 | 55.9 ± 7.8 | — | — | 0.979 |
|  | 12 weeks | 57.9 ± 11.2 | 57.0 ± 7.0 | −0.8 (1.5) | [−3.9, 2.2] | 0.587 |
| Executive function | Baseline | 41.9 ± 12.1 | 43.6 ± 12.4 | — | — | 0.582 |
|  | 12 weeks | 45.4 ± 11.2 | 46.9 ± 13.2 | 0.2 (1.9) | [−3.6, 4.0] | 0.922 |
| Social acuity | Baseline | 7.0 ± 2.6 | 6.2 ± 2.7 | — | — | 0.214 |
|  | 12 weeks | 7.1 ± 2.5 | 6.5 ± 2.6 | −0.3 (0.6) | [−1.5, 0.9] | 0.646 |
| Reasoning | Baseline | 6.6 ± 3.7 | 5.2 ± 4.2 | — | — | 0.145 |
|  | 12 weeks | 5.6 ± 4.5 | 6.2 ± 4.8 | 1.6 (0.9) | [−0.2, 3.4] | 0.077 |
| Working memory | Baseline | 9.7 ± 3.6 | 10.0 ± 3.9 | — | — | 0.719 |
|  | 12 weeks | 10.5 ± 2.9 | 10.5 ± 3.8 | −0.1 (0.7) | [−1.6, 1.3] | 0.850 |

(Continued)

**Supplementary Table S1** Continued

| **Outcome** | **Time point** | **Placebo (n = 33)** | **sPls (n = 33)** | **Difference (SE)** | **95% CI** | ***p*-value** |
| --- | --- | --- | --- | --- | --- | --- |
| Sustained attention | Baseline | 29.3 ± 6.1 | 28.6 ± 6.8 | — | — | 0.689 |
|  | 12 weeks | 31.5 ± 3.2 | 29.8 ± 7.8 | −1.4 (1.3) | [−4.1, 1.2] | 0.280 |
| Simple attention | Baseline | 38.0 ± 4.8 | 38.9 ± 2.4 | — | — | 0.321 |
|  | 12 weeks | 38.9 ± 1.1 | 39.5 ± 0.9 | 0.5 (0.2) | [0.0, 1.0] | 0.037* |
| Motor speed | Baseline | 103.6 ± 18.0 | 112.3 ± 14.6 | — | — | 0.034 |
|  | 12 weeks | 107.2 ± 12.5 | 113.5 ± 13.6 | 0.5 (1.9) | [−3.3, 4.3] | 0.792 |

Values are mean ± SD. Differences are expressed as sPls minus placebo. Between-group differences were estimated using analysis of covariance (ANCOVA) with baseline values as covariates. Baseline *p*-values were calculated using Welch’s t-test. Within-group comparisons were performed using paired t-tests. SD, standard deviation; SE, standard error; CI, confidence interval; sPls, scallop-derived plasmalogens. NA: Not Available. * *p* < 0.05, ** *p* < 0.01, *** *p* < 0.001.

**Supplementary Table S2** Pearson’s correlation between plasma BDNF levels and standardized Cognitrax scores

| Cognitive domain | BDNF | | | | |
| --- | --- | --- | --- | --- | --- |
|  | Placebo | |  | sPls | |
|  | Baseline | 12 weeks |  | Baseline | 12 weeks |
| Neurocognitive index | 0.191 | 0.149 |  | 0.074 | −0.158 |
| Composite memory | 0.266 | 0.017 |  | −0.085 | −0.337 |
| Verbal memory | 0.114 | −0.034 |  | 0.042 | **−0.374** |
| Visual memory | **0.390** | 0.067 |  | −0.153 | −0.133 |
| Psychomotor speed | 0.298 | 0.044 |  | 0.129 | −0.132 |
| Reaction time | 0.113 | 0.112 |  | 0.020 | −0.143 |
| Complex attention | −0.155 | 0.269 |  | 0.112 | 0.083 |
| Cognitive flexibility | 0.107 | 0.145 |  | 0.067 | 0.094 |
| Processing speed | 0.072 | −0.110 |  | 0.003 | −0.141 |
| Executive function | 0.087 | 0.100 |  | 0.053 | 0.095 |
| Social acuity | 0.084 | 0.069 |  | 0.039 | 0.125 |
| Reasoning | 0.061 | 0.063 |  | −0.326 | −0.286 |
| Working memory | −0.020 | −0.064 |  | −0.329 | **−0.478** |
| Sustained attention | 0.129 | −0.001 |  | **−0.447** | **−0.440** |
| Simple attention | −0.276 | 0.025 |  | 0.039 | 0.161 |
| Motor speed | 0.334 | 0.159 |  | 0.161 | −0.108 |

The darker color indicates that the correlation coefficient increases gradually. For example, red shows a positive correlation, while blue shows a negative correlation. The values indicate Pearson’s correlation coefficients (*r*), and bold letters indicate *p* < 0.05. sPls, scallop-derived plasmalogens; BDNF, brain-derived neurotrophic factor.

**Supplementary Table S3** Summary of abnormal laboratory and urinalysis findings after intervention

| **Parameter** | **Placebo (n = 33) n (%)** | **sPls (n = 33) n (%)** | **Difference (%)** | **95% CI** | ***p*-value** |
| --- | --- | --- | --- | --- | --- |
| Urinalysis findings | 3 (9.1) | 2 (6.1) | −3.0 | [−15.8, 9.7] | 1.000 |
| Hematological parameters | 5 (15.2) | 4 (12.1) | −3.0 | [−19.6, 13.5] | 1.000 |
| Liver function markers | 3 (9.1) | 2 (6.1) | −3.0 | [−15.8, 9.7] | 1.000 |
| Renal function markers | 5 (15.2) | 6 (18.2) | 3.0 | [−14.9, 21.0] | 1.000 |
| Electrolyte abnormalities | 1 (3.0) | 1 (3.0) | 0.0 | [−8.3, 8.3] | 1.000 |
| Lipid metabolism markers | 4 (12.1) | 6 (18.2) | 6.1 | [−11.2, 23.3] | 0.733 |
| Glucose metabolism markers | 1 (3.0) | 2 (6.1) | 3.0 | [−7.0, 13.1] | 1.000 |

Values are number of participants (%). Differences are expressed as sPls minus placebo. Group comparisons were performed using Fisher’s exact test.

Participants were counted once per category if any parameter within that category exceeded the reference range. All analyses were conducted on a complete-case basis. CI, confidence interval; sPls, scallop-derived plasmalogens.
